# Prediabetes in acute coronary syndrome: an overlooked predictor of adverse outcomes

**DOI:** 10.3389/fcdhc.2026.1745724

**Published:** 2026-03-12

**Authors:** Daniel Hakim, Louay Taha, Mohammad Karmi, Eyal Ben-Zvi, Asher Schnur, Rafael Hitter, Noam Fink, Pierre Sabouret, Mamas A. Mamas, Ranim Aouda, Akiva Brin, Ari Naimark, Amjad Abu-Salaman, Michael Glikson, Elad Asher

**Affiliations:** 1 Jesselson Integrated Heart Center, The Eisenberg R&D Authority, Shaare Zedek Medical Center and Faculty of Medicine, Hebrew University of Jerusalem, Jerusalem, Israel; 2 Assuta Medical Centers, Israel, Faculty of Medicine, Hebrew University of Jerusalem, Jerusalem, Israel; 3 Alliance for Clinical Trials in Cardiology and Cardiovascular Diseases (ACTION) Study Group, Institut de Cardiologie, Hôpital Pitié-Salpêtrière, Sorbonne Université, Paris, France; 4 Department of Cardiology, National College of French Cardiologists, Paris, France; 5 Keele Cardiovascular Research Group, Centre for Prognosis Research, Keele University, Stoke-on-Trent, United Kingdom; 6 Department of Cardiothoracic Surgery, Shaare Zedek Medical Center and Faculty of Medicine, Hebrew University of Jerusalem, Jerusalem, Israel

**Keywords:** acute coronary syndrome (ACS), diabetes mellitus, HbA1c - hemoglobin A1c, mortality, NSTEMI = non ST elevation myocardial infarction, STEMI (myocardial infarction)

## Abstract

**Background:**

Hemoglobin A1c (HbA1c) is a well-established marker for long-term glycemic control and a diagnostic tool for diabetes mellitus (DM). The relationship between HbA1c levels and prognosis among acute coronary syndrome (ACS) patients is not well described. The aim of the current study was to assess HbA1c levels as an independent predictor of mortality in patients with ACS admitted to contemporary intensive cardiovascular care unit (ICCU).

**Methods:**

A retrospective single center study included all patients admitted to the ICCU between July 2019 and December 2024 with ACS. Patients were categorized by HbA1c levels into three groups: non-DM (<5.7%), pre-DM (5.7–6.4%), and DM (≥6.5%). Demographics, clinical characteristics, in-hospital complications, and long-term (up to 60 months) mortality were analyzed.

**Results:**

A total of 2,772 patients were admitted with a diagnosis of ACS and had HbA1c levels recorded at admission. Among them, 41.4% were non-diabetic, 29.1% had pre-diabetes, and 29.5% had diabetes. In-hospital mortality showed a gradual increase across these groups: 2.0% in non-diabetics, 1.6% in pre-diabetics, and 2.9% in diabetics (p = 0.294). Long-term mortality rose significantly with higher HbA1c categories, reaching 11.4%, 14.7%, and 18.1%, respectively (p<0.001). Multivariate analysis confirmed DM as an independent predictor of mortality (HR 1.635, 95% CI: 1.280–2.08, p<0.001).

**Conclusions:**

Over half of patients admitted with an ACS have evidence of dysglycemia. Both pre-DM and DM groups were associated with increased long-term mortality in ACS patients. The findings highlight the need for greater recognition and management, especially of pre-DM ACS patients in acute cardiovascular care.

## Introduction

Despite advances in therapeutic strategies, acute coronary syndromes (ACS) continue to be associated with significant morbidity and mortality. According to the American Heart Association (AHA), approximately 18% of men and 23% of women over the age of 40 will die within one year of experiencing an acute myocardial infarction (MI) (1, 2).

Established risk factors for ACS include cigarette smoking, hypertension, hyperlipidemia, and diabetes mellitus (DM). Increasingly patients with pre-DM are recognized as having an increased risk of cardiovascular disease and may experience higher morbidity and mortality rates compared to their non-diabetic counterparts (3).

Hemoglobin A1c (HbA1c), a form of glycated hemoglobin, is commonly used to assess long-term glycemic control in individuals with DM and can be helpful in identifying patients that are pre-DM. Elevated baseline HbA1c levels have been associated with increased mortality (4). In patients presenting with ACS, acute glycemic status—assessed by plasma glucose levels—may be a more accurate predictor of prognosis than chronic glycemic control, as estimated by HbA1c.

Currently, data is limited regarding the relationship between pre-DM HbA1c levels and ACS prognosis (4–6). The JUPITER-6 study demonstrated that patients with pre-DM HbA1c levels had worse overall prognoses than both DM and non-DM patients, this is likely attributable to the fact that pre-DM patients did not receive treatment in accordance with American Heart Association (AHA) guidelines, whereas patients with diagnosed DM were managed appropriately for their condition. A phenomenon referred to as the “prediabetic paradox” (7).

Hence, the aim of the current study was to evaluate the prognostic significance of admission HbA1c levels, particularly within the pre-DM range, among patients with ACS admitted to a tertiary care medical center Intensive Cardiovascular Care Unit (ICCU).

## Methods

### Study population

A Retrospective single-center study at the Shaare Zedek Medical Center, a tertiary referral hospital and one of the 2 largest medical centers in Jerusalem. The study population consisted of consecutive ACS patients admitted to the ICCU between 1 July 2019 and 31 December 2024.

#### Inclusion criteria

Patients were included if they were admitted due to ACS and at the same time had HbA1c levels taken on admission.

#### Exclusion criteria

Patients without a confirmed diagnosis of ACS, with out-of-hospital sudden death (OHSD) and/or type II myocardial infarction and periprocedural MI were excluded.

#### HbA1c levels

Patients were divided into 3 groups according to their HbA1c levels: < 5.7g% (no DM), 5.7–6.4g% (pre-DM) and ≥ 6.5g% (DM) according to contemporary guidelines (8).

The assay that was used was Abbott ARCHITECT HbA1c. Obesity was defined as a body mass index (BMI)>30. Demographic data, comorbid conditions, medications, physical examination, laboratory findings, in-hospital complications, length of stay (LOS), and in-hospital and long term (up to 60 months) mortality were recorded as well. Pharmacological therapy was administered according to institutional protocols and international guidelines. Specifically, SGLT2 inhibitor therapy consisted of Empagliflozin 10mg daily, and GLP-1 receptor agonist therapy followed standard titration starting at 0.25mg, as per FDA dosing recommendations.

### Study outcome

The primary outcome was long term overall mortality and complications during admission. Mortality status was derived from the central database of the Israeli Ministry of the Internal Affairs.

In addition, patients were further categorized into two clinical subgroups according to ACS subtype: ST-elevation myocardial infarction (STEMI) and non-ST-elevation myocardial infarction (NSTEMI), which included NSTEMI and unstable angina pectoris (UAP) according to contemporary guidelines (9–11).

Furthermore, we conducted a survival analysis for both STEMI and NSTEMI cohorts, further subdividing each according to glycemic status as defined by HbA1c levels.

Ethics:

This study complied with the Declaration of Helsinki and has been approved by the Institutional Review Board (IRB) at the Shaare Zedek Medical Center (IRB protocol number 0330-24-SZMC). The trial was not funded by any external source. Informed consent was waived by the IRB due to the observational design of the study.

### Statistical methods

#### Sample size

Sample size was based on the expected difference in mortality within 3 years among patients with ACS, between patients without DM, pre DM and with DM. Based on that mortality rates will be 8%, 12%, 13% respectively (7), that 40% of the patients will be non DM, 32% pre DM and 28% with DM, the significance level is 5%, and at least 1800 patients with ACS will be included in the study. The sample size was calculated using the Cochran formula, as applied in similar study designs. there will be a power of 80% to prove that the difference between the groups is statistically significant.

#### Data analysis

Testing the effect of categorical variables on survival (e.g. the effect DM status (non-DM pre-DM and DM) on survival) was performed by using the Kaplan-Meier Survival analysis method with the log-rank test for the comparison of survival curves. Testing the effect of quantitative variables (e.g. age) on survival was performed using the Cox regression model. This model was applied as the multivariate model for survival.

Testing association between two categorical variables (e.g. DM status and smoking) was performed by applying the Chi-square test.

Comparing quantitative variables between 3 independent groups (e.g. age between the 3 DM status groups) was performed by using the ANOVA test, with post-Hoc test, while applying the correction of the p- value for multiple, pairwise, comparisons.

## Results

**Figure 1:**
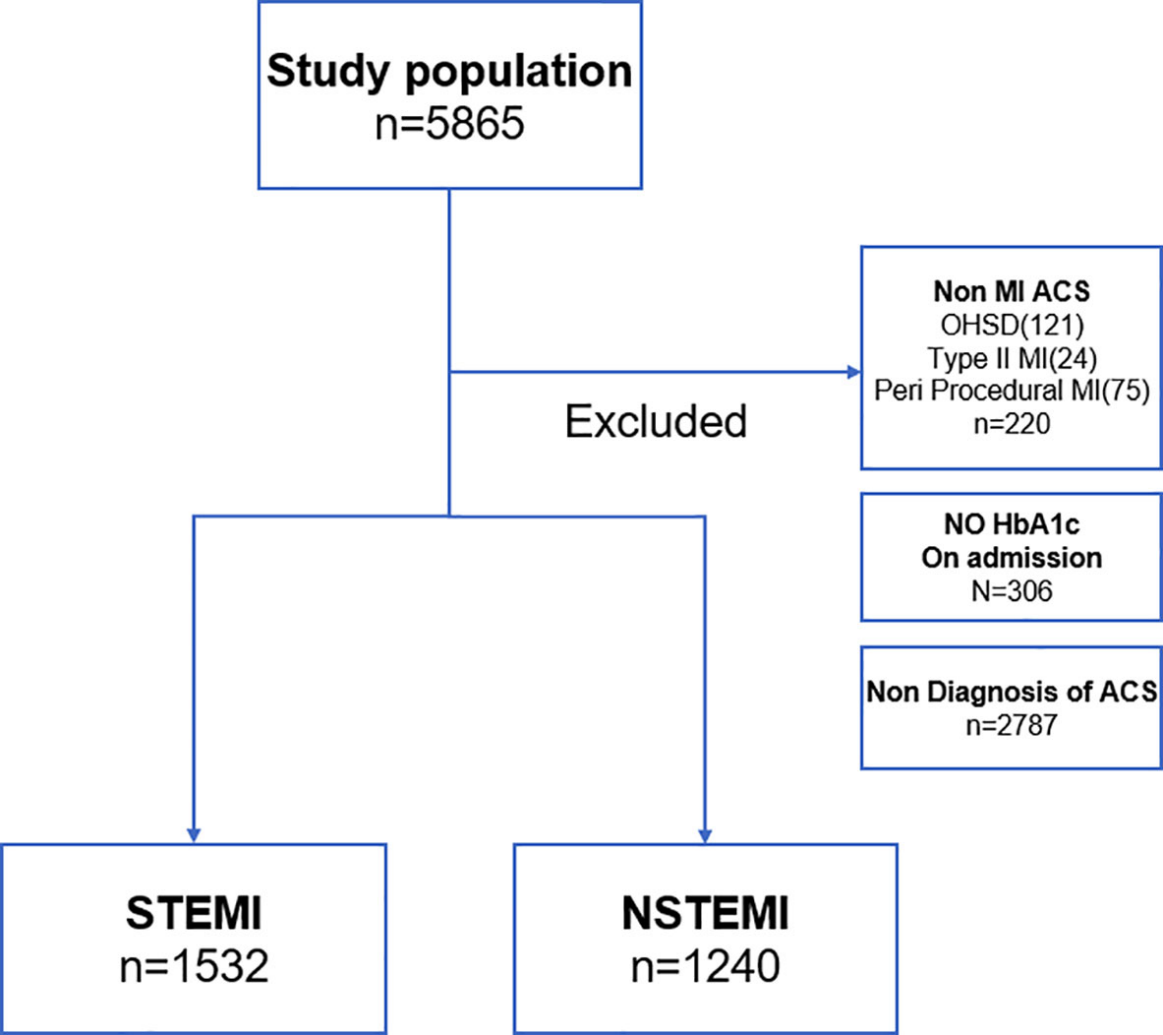
Patients enrollment scheme. ACS, acute coronary syndrome; OHSD, out of hospital sudden death; MI, myocardial infarction; STEMI, ST segment elevation myocardial infarction; NSTEMI, non ST segment elevation myocardial infarction.

A total of 5,865 patients were admitted during the study period, of whom 5,559 (95%) had HbA1c levels assessed on admission. Among these, 2,772 (50%) patients were diagnosed with ACS and formed the study population, including 1,532 (55%) with STEMI and 1,240 (45%) with NSTEMI, as illustrated in **Figure 1**. Within this cohort, 1,147 (41.4%) patients were classified as non-diabetic, 807 (29.1%) as pre-diabetic, and 818 (29.5%) as diabetic. Importantly, 179 patients (6.5%) had admission HbA1c levels ≥6.5%, consistent with diabetes, despite no prior diagnosis.

### Patients’ characteristics

The mean age of the cohort was 65.5 ± 13.6 years, and 635 patients (23%) were female. Patients in the non-DM group were significantly younger than those in the pre-DM and DM groups, with mean ages of 63.5 years (95% CI: 62.65–64.37), 67.3 years (95% CI: 66.38–68.18), and 66.6 years (95% CI: 65.75–67.37), respectively (p < 0.001). Furthermore, prior ACS was more prevalent among patients in the pre-DM and DM groups All participants underwent at least one transthoracic Echo assessment to determine left ventricular ejection fraction (LVEF). Moreover, 409 patients underwent diagnostic coronary angiography and 2136 patients underwent percutaneous coronary intervention (PCI), as detailed in **Table 1**.

**Table 1:** Demographic and baseline characteristics of patients stratified by HbA1c.

Variables	groups			
	non-DM 1147 (41.4%)	pre-DM 807 (29.1%)	DM 818 (29.5%)	p-value
Age, mean	63.51{62.65-64.37}	67.28{66.38-68.18}	66.56{65.75-67.37}	0.016
BMI, mean (SD)	27.14 {4.64}	28.38 {5.3}	28.67 {4.99}	0.019
Female gender	257{40.5%}	187{29.4%}	191{30.1%}	0.867
History of prior ACS	285{32.4%}	263 {29.9%}	332{37.7%}	0.001
Cath	166{14.5%}	120{14.9%}	123{15%}	0.936
PCI	877{76.5%}	631{78%}	628{76.8%}	0.652
HTN	547{33.8%}	495{30.6%}	576{35.6%}	0.001
DLP	547{34.3%}	465{29.1%}	585{36.6%}	0.001
EF, Mean (SD)	48.37{11.33}	48.34{11.83}	46.7{10.3}	0.002
Smoking	442{43.6%}	281{27.7%}	291{28.7%}	0.19

DM, Diabetes Mellitus; BMI, Body Mass Index; ACS, Acute coronary syndrome; PCI, Percutaneous Coronary Intervention; HTN, Hypertension; DLP, Dyslipidemia; EF, Ejection Fraction.

### In−hospital complications and mortality rates

Overall complication rates were comparable across all groups (p = 0.683), with left ventricular thrombus and acute renal failure being the most frequent events. In-hospital mortality was 2.0% in non-DM patients, 1.6% in pre-DM patients, and 2.9% in DM patients (p = 0.294) (**Table 2**).

**Table 2:** In hospital complications (* by percentage).

Complications	non-DM 1147 (41.4%)	pre-DM 807 (29.1%)	DM 818 (29.5%)	P-value
Malignant arrhythmia	2.5	2.5	2	0.680
Shock	3.4	2.2	3.1	0.317
Heart failure	3.1	2.7	4	0.294
Mechanical complications-VSR\rupture	0.7	0.5	0.7	0.808
LV thrombus	1.5	2.4	1.2	0.168
Sepsis	1.7	1.4	2.2	0.419
Stroke\TIA	0.5	0.2	0.6	0.529
Re-infraction	1	0.9	0.6	0.697
Stent thrombosis	0.1	0	0.1	0.637
Acute renal failure	2.4	3.6	3.8	0.176
Significant bleeding	1.8	1.9	2.4	0.589
Blood transfusion	2.5	2.6	2.6	0.995
Vascular	1.1	1.1	0.9	0.815
Anoxic Brain damage	0.7	0.2	0.9	0.26
Mortality	2	1.6	2.9	0.294
Number of complications	0.23	0.22	0.25	0.683

LV, Left Ventricle; TIA, Transient ischemic attack; VSR, Ventricular septal rupture.

### Long-term mortality rate

**Figure 2:**
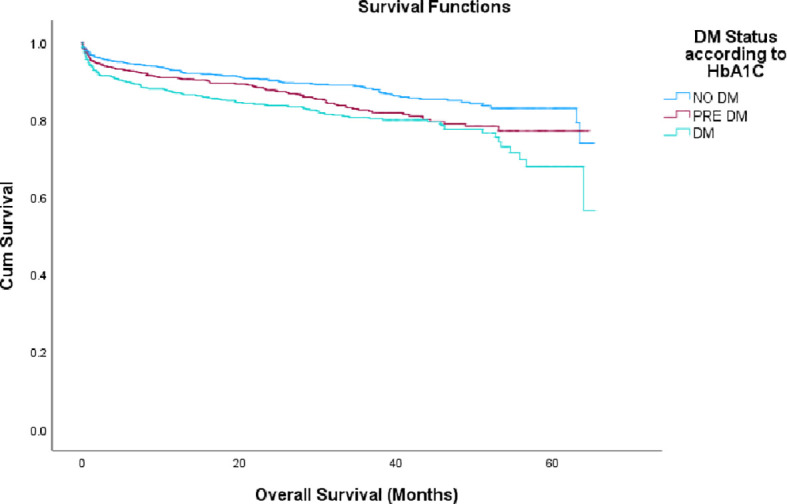
Mortality rate by months according to HbA1c groups. DM, diabetes mellitus; HbA1c, glycated hemoglobin.

Cumulative mortality rates were increased progressively across glycemic categories: 11.4% vs. 14.7% vs. 18.1% in patients with non-DM, pre-DM and DM, respectively, (p<0.001), as illustrated in **Figure 2**.

After multivariate analysis patients with DM exhibited the strongest association with mortality among all groups, with a HR of 1.635 (95% CI: 1.28–2.08; p<0.001), while the pre-DM group did not reach statistically significant with a HR of 1.179 (95% CI: 0.913–1.542; p=0.206). Additional variables found to be independently associated with increased mortality included advanced age, the number of in-hospital complications, and a prior history of ACS (either STEMI or NSTEMI), as detailed in **Table 3**.

**Table 3:** Multivariate Cox-logistic regression model for mortality including: Age Gender, STEMI, NSTEMI, HTN, DLP, Smoking and DM status, history of ACS and number of complications.

Variables	HR	95% CI	p-value
Number of complications	2.311	1.762-3.031	<0.0001
Age	1.054	1.045-1.063	<0.001
BMI	0.974	0.952-0.997	0.026
History of ACS	1.698	1.387-2.079	0.001
HbA1c Between 5.7-6.4%	1.179	0.913-1,524	0.206
HbA1c >6.5%	1.635	1.280-2.088	<0.0001
STEMI	1.374	1.165-1.622	<0.0001
NSTEMI	1.468	1.241-1.736	<0.0001

BMI, Body mass index; ACS, Acute Coronary Syndrome; HbA1c, Hemoglobin A1c; STEMI, ST-Elevation Myocardial Infarction; NSETMI, Non-ST-Elevation Myocardial Infarction.

### STEMI vs. NSTEMI

**Figure 3:**
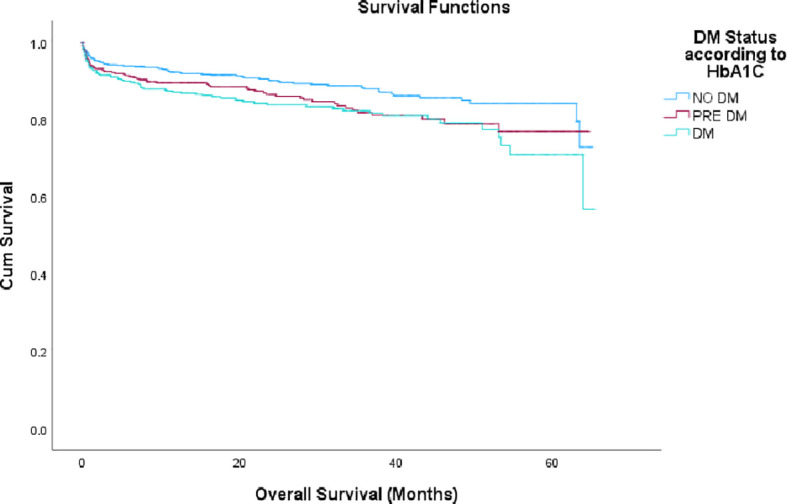
Mortality rate by HbA1c groups in STEMI patients. STEMI, ST segment elevation myocardial infarction; DM, diabetes melitus; HbA1c, glycated hemoglobin.

Patients with STEMI also showed increased cumulative long term mortality rates across glycemic categories: 11.3% vs. 14.9% vs. 17.3% in patients with non-DM, pre-DM and DM, respectively, (p=0.002), as shown in **Figure 3**.

**Figure 4:**
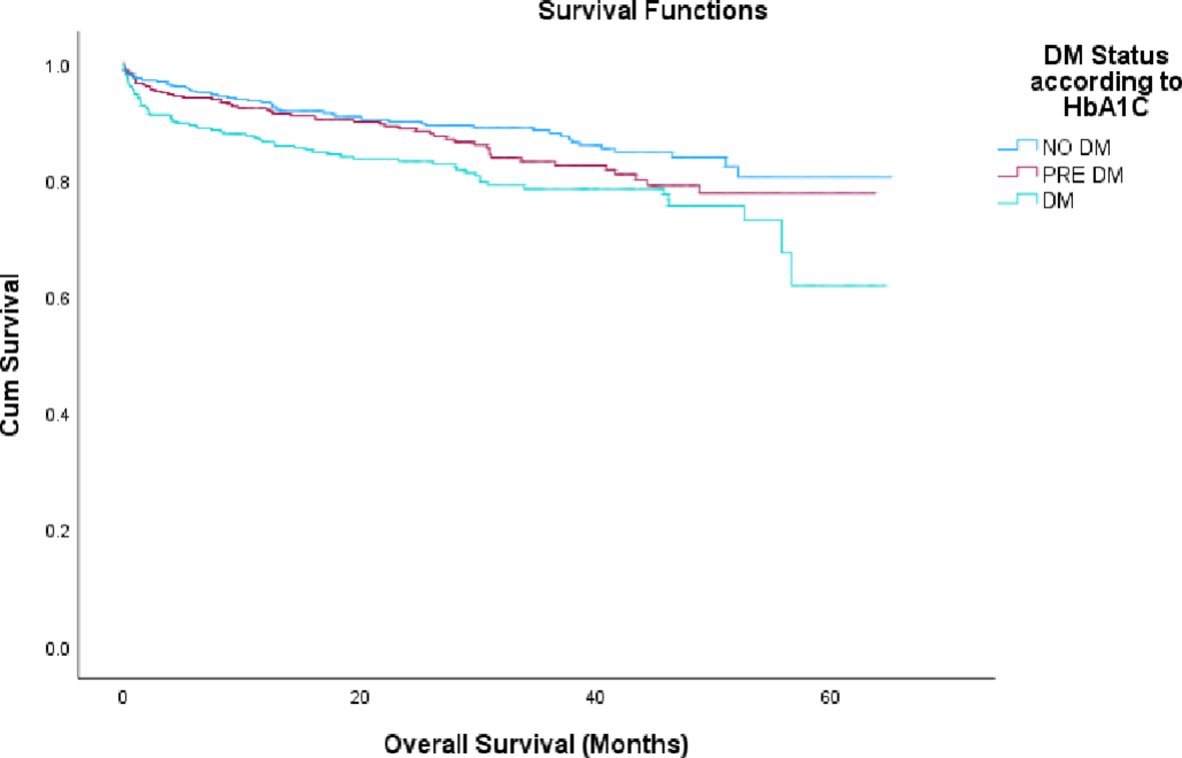
Mortality rate by HbA1c groups in NSTEMI patients. NSTEMI, non-ST segment elevation myocardial infarction; DM, diabetes melitus; HbA1c, glycated hemoglobin.

Furthermore, in the NSTEMI group the long-term mortality rates were higher than the STEMI group with 11.6% vs. 14.6% vs. 19.1% in patients with non-DM, pre-DM and DM, respectively, (p=0.001), as shown in **Figure 4**.

## Discussion

The present Retrospective study, conducted in a large cohort of 2772 consecutive ACS patients admitted to a tertiary care center ICCU, demonstrates that over half of patients had evidence of dysglycemia, with almost one third of patients having HbA1c measurements suggesting pre-diabetes and one third of patients in which HbA1c measurements fell within the diabetic range. Furthermore, our findings indicate that both pre-DM and DM were associated with an increased risk of long-term mortality; however, after adjusting for key covariates such as age, dyslipidemia, prior ACS, and in-hospital complications, only the DM group consistently demonstrated this association in both STEMI and NSTEMI subgroups.

Our findings add to the growing body of evidence that dysglycemia—even in the range below the diagnostic threshold for DM—is a significant and independent determinant of adverse cardiovascular outcomes following ACS (12, 13). While the elevated mortality risk in patients with DM is well established (14), the recognition of pre-DM as a clinically relevant risk marker is relatively recent (15). In this study, pre-DM patients exhibited a hazard ratio for mortality of 1.236 compared with non-DM patients, and DM patients had an even higher hazard ratio of 1.554. Importantly, mortality increased in a stepwise fashion across the glycemic spectrum, underscoring the dose-response relationship between chronic hyperglycemia and adverse prognosis.

### Pre-diabetes and the “Prediabetic Paradox”

Prediabetes represents a transitional state characterized by impaired fasting glucose or impaired glucose tolerance, reflected here by HbA1c levels between 5.7–6.4%. This stage is associated with metabolic and vascular abnormalities that often precede the onset of overt diabetes by several years (16, 17). Emerging literature, including the present findings, challenges the traditional perception that pre-DM is a relatively benign state. Instead, it is increasingly recognized as a period of heightened cardiovascular risk—one in which patients are often undiagnosed, untreated, and thus unprotected from targeted interventions (18).

The concept of the “prediabetic paradox” may help explain our results. Individuals with diagnosed DM typically receive structured follow-up, pharmacologic treatment (including cardioprotective agents such as SGLT2 inhibitors and GLP-1 receptor agonists), and lifestyle counseling (19). In contrast, pre-DM patients often remain unrecognized in acute care setting and are not systematically enrolled in secondary prevention programs. This therapeutic gap may contribute to their elevated mortality risk, which in some series approaches or even exceeds that of DM patients. In our cohort, pre-DM patients had mortality rates closer to the DM group than to the non-DM group, reinforcing the urgency of addressing this overlooked population.

### STEMI and NSTEMI subgroup analysis

Our study also demonstrates that the adverse impact of dysglycemia is not confined to a particular ACS phenotype (20). Both STEMI and NSTEMI patients with pre-DM or DM had higher long-term mortality compared with normoglycemic counterparts. Interestingly, the absolute mortality rates were higher in the NSTEMI subgroup across all glycemic strata. This may reflect the older age, higher comorbidity burden, and more diffuse coronary artery disease typically observed in NSTEMI patients (21). Nonetheless, the relative risk conferred by pre-DM and DM remained significant in both groups, suggesting that glycemic status exerts a uniform pathophysiological influence on post-ACS outcomes (22).

The mechanism underlying these associations is likely multifactorial. Chronic hyperglycemia promotes endothelial dysfunction, systemic inflammation, oxidative stress, and prothrombotic states—all of which contribute to accelerated atherosclerosis and plaque instability (23). Additionally, pre-DM and DM are associated with adverse myocardial remodeling, autonomic dysfunction, and impaired microvascular function, which may exacerbate post-infarction heart failure and arrhythmic risk (24).

### Clinical implications

The present findings have several important clinical implications. First, routine measurement of HbA1c in ACS patients—regardless of prior DM diagnosis—can help identify individuals at increased long-term risk. Our data show that nearly 15.6% of patients had HbA1c ≥6.5% without a previous DM diagnosis, emphasizing the role of ACS hospitalization as an opportunity for detection.

Second, the identification of pre-DM should trigger more aggressive risk factor modification. Current guidelines emphasize lifestyle intervention for pre-DM but do not recommend pharmacologic therapy as a standard. However, given the magnitude of risk observed here, a reevaluation of this approach may be warranted, at least in the high-risk setting of post-ACS care. This could include closer follow-up, early initiation of cardioprotective pharmacotherapy, and integration into cardiac rehabilitation programs (12, 25).

Third, our results suggest that pre-DM and DM patients should be considered high-priority targets for comprehensive secondary prevention strategies, including stringent control of blood pressure, lipids, and body weight, as well as smoking cessation and optimal revascularization strategies.

### Study limitations

Several limitations should be acknowledged.

1. Observational and single-center design – Although the study was Retrospective and included a large consecutive cohort, the single-center setting may limit generalizability to other healthcare systems and populations.
2. Residual confounding – Despite multivariate adjustment, unmeasured variables such as socioeconomic status, dietary patterns, medication adherence, and genetic predispositions may have contributed to the results.
3. Absence of detailed treatment data – We did not capture information on subsequent antidiabetic therapy, intensity of secondary prevention, or adherence to lifestyle recommendations. This limits our ability to assess whether differences in treatment intensity between groups contributed to mortality outcomes.
4. Data regarding acute glycemic fluctuations, such as admission or fasting glucose levels, were not collected. While this may limit the assessment of acute metabolic stress, the study focused on HbA1c as a validated measure of long-term glycemic management. We believe that HbA1c provides a more stable reflection of the patients’ baseline metabolic state, though we recognize that the absence of acute glucose data remains a limitation in the interpretation of immediate clinical responses.

## Conclusions

This study demonstrates that both pre-DM and DM are associated with increased long-term mortality in ACS patients, regardless of whether they present with STEMI or NSTEMI. Mortality risk increases progressively across HbA1c categories, supporting the concept of a continuum of cardiovascular risk linked to chronic dysglycemia.

The findings underscore the importance of systematic HbA1c measurement in ACS admissions and the need to address pre-DM as an active cardiovascular risk state, not merely a precursor to diabetes. Early identification and aggressive secondary prevention strategies in both pre-DM and DM patients may offer an opportunity to reduce the substantial burden of post-ACS mortality.

Future multicenter, randomized studies are needed to determine whether targeted pharmacologic intervention in pre-DM ACS patients can modify long-term outcomes, and to explore the optimal combination of lifestyle and pharmacologic strategies for this high-risk population.

## Data Availability

The raw data supporting the conclusions of this article will be made available by the authors, without undue reservation.

## Author's note

This work was conducted in partial fulfillment of the requirements for the degree of Doctor of Medicine (MD) at the Hebrew University–Hadassah School of Medicine.
